# The Association of Maternal Pre-Pregnancy Overweight with Childhood BMI Trajectories and Blood Pressure

**DOI:** 10.3390/healthcare14111487

**Published:** 2026-05-27

**Authors:** Tianshu Feng, Rui Deng, Weiqin Li, Changyuan Zhou, Jie Hu, Jing Li, Bin Dong

**Affiliations:** 1 Institute of Child and Adolescent Health, School of Public Health, Peking University Health Science Center, Beijing 100191, China; 2211110225@stu.pku.edu.cn (T.F.);; 2 Institute of Nutrition and Food Hygiene, Beijing Center for Disease Prevention and Control, Beijing 100013, China; 3 Tianjin Women and Children’s Health Center, No. 96 Guizhou Rd., Heping District, Tianjin 300070, China; 4 School of Pharmacy and Medical Sciences, Griffith University, Nathan, QLD 4111, Australia

**Keywords:** childhood, high blood pressure, maternal pre-pregnancy overweight, early-life BMI trajectories

## Abstract

**Highlights:**

- Early BMI trajectories may exert a probable modulating effect on the association between maternal pre-pregnancy overweight and childhood high blood pressure (HBP).
- Maternal pre-pregnancy overweight and rapid early-life BMI growth jointly contribute to elevated risk of HBP.
- Children with both maternal pre-pregnancy overweight/obesity and rapid early-life BMI growth represent a priority target group for childhood HBP prevention and intervention.

**Abstract:**

**Background**: Childhood high blood pressure (HBP) represents a significant risk for long-term cardiovascular health. However, the role of body mass index (BMI) trajectories from birth in shaping early-life blood pressure (BP) remains poorly understood. **Methods**: Data derived from a prospective birth cohort comprising 886 children (433 boys), followed from maternal pregnancy to a mean age of 9.11 years (SD = 0.71), were analyzed. Group-based trajectory modeling (GBTM) was used to identify distinct childhood BMI trajectories. Binomial regression models were applied to examine the associations among maternal pre-pregnancy overweight, childhood BMI trajectories, and the risk of HBP. Mediation analysis was used to examine the potential modulating role of BMI trajectories in the association between maternal pre-pregnancy overweight and childhood HBP. **Results**: Five distinct BMI trajectories were identified. Children in the “persistent overweight” (RR = 2.52, 95% CI: 1.50–4.26, *p* < 0.001) and “persistent obesity” (RR = 3.34, 95% CI: 1.76–6.36, *p* < 0.001) trajectories demonstrated significantly increased risks of HBP (The “stable normal weight” group, RR = 1). Mediation analysis revealed that BMI trajectories were associated with the linkage between maternal pre-pregnancy overweight and offspring HBP, with an indirect effect size of 0.029 (95% CI: 0.017–0.040), accounting for 34.9% of the total effect (*p* < 0.05). Furthermore, among children exposed to maternal pre-pregnancy overweight, those in the persistent overweight (RR = 3.35, 95% CI: 1.57–7.12, *p* < 0.001) and persistent obesity (RR = 4.02, 95% CI: 1.60–10.08, *p* < 0.001) trajectories exhibited particularly strong associations with HBP. **Conclusions**: Maternal pre-pregnancy overweight was linked to the elevated risk of childhood HBP, and childhood BMI trajectories may be associated with the linkage.

## 1. Introduction

Although high blood pressure (HBP) is typically diagnosed in adults, its prevalence among children has increased substantially over the past two decades [1]. In children, HBP can result in early target organ damage, including cardiac, cerebral, and renal impairment [2]. Moreover, the rising prevalence of childhood HBP has been linked to an increased risk of persistent HBP in adulthood [3,4,5]. Therefore, early identification of at-risk populations and timely intervention are crucial for mitigating long-term cardiovascular burden.

Current research has examined numerous risk factors for childhood HBP. However, few studies have focused on the impact of maternal pre-pregnancy and prenatal health on offspring blood pressure (BP) during childhood. Emerging evidence suggests that maternal pre-pregnancy overweight may contribute to adverse outcomes in offspring [6]. In addition, both inadequate and excessive maternal gestational weight gain (GWG) have been linked to unfavorable early-life outcomes [7,8]. Nevertheless, the impact of maternal pre-pregnancy overweight and GWG on childhood HBP remains insufficiently understood. It is possible that these maternal factors may also influence offspring BP.

Our previous work found that maternal pre-pregnancy overweight and excessive GWG were associated with rapidly increasing, relatively high BMI trajectories in offspring [8]. Distinct childhood BMI trajectories have also been linked to childhood HBP. Some studies have shown that consistently high or rapidly increasing BMI trajectories in children are associated with an increased risk of childhood HBP [9]. Building on these observations, we hypothesize that maternal pre-pregnancy overweight and excessive GWG may be associated with offspring HBP, and childhood BMI trajectories play an important role in the associations.

Furthermore, although maternal pre-pregnancy overweight, excessive GWG, and rapid BMI increase in children have each been identified as independent risk factors for adverse health outcomes, their combined effects remain inadequately explored. Therefore, we aimed to examine the combined effects of maternal pre-pregnancy overweight and offspring BMI trajectories on childhood HBP. To address these knowledge gaps, we conducted a longitudinal birth cohort study tracking participants from maternal pre-pregnancy to 9–11 years of age. The aims of this study were to: (1) assess the effects of maternal pre-pregnancy overweight, maternal GWG, and offspring BMI trajectories on childhood HBP, (2) examine whether BMI trajectories are associated with the linkage between maternal pre-pregnancy overweight (and GWG) and childhood HBP, and (3) investigate the combined influence of pre-pregnancy overweight (and GWG) and offspring BMI distinct trajectories on childhood HBP.

## 2. Materials and Methods

### 2.1. Data Source

**Figure A1:**
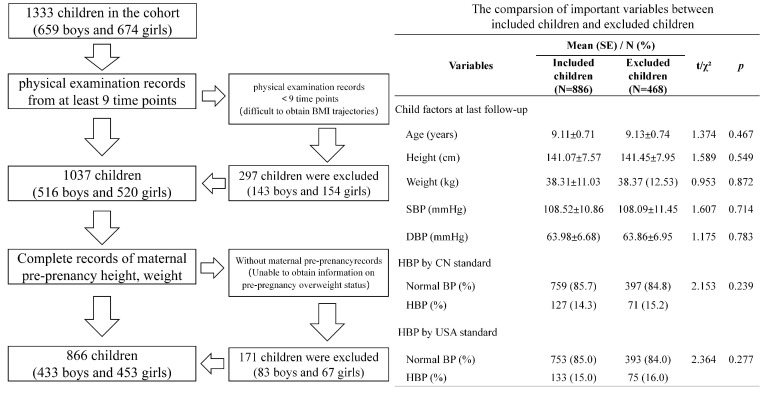
The flowchart of participant selection. Notes: The variables were compared between included children and excluded children.

A cluster sampling strategy was employed to recruit all students in grades 1–2 from three elementary schools in Tianjin, China. Data collected prior to school enrollment were obtained from the Tianjin Women and Children’s Health Center. These records included general maternal information, clinical measurements, and data from twelve physical examinations of the offspring conducted from birth through preschool age. To ensure data completeness, children were required to have physical examination records for at least 9 time-points, including mandatory measurements of height and weight at birth and at the final follow-up assessment. Based on these criteria, 296 children were excluded. Additionally, complete records of maternal pre-pregnancy height, weight, and BP were required; an additional 171 children were excluded due to missing maternal information. A flowchart of participant selection is presented in Appendix A (Figure A1). No significant differences in height, weight, and BP at the last survey were observed between the excluded and included children. Ultimately, a total of 886 children were included in the final analysis (mean age = 9.11 years, standard deviation [SD] = 0.71), as detailed in Table 1. Following school entry, participants underwent annual physical examination and completed self-administered questionnaires at each visit. These data were linked to early-life healthcare records through a unique healthcare identification number assigned to each child.

### 2.2. Maternal Measurements and GWG

Maternal pre-pregnancy weight and height were obtained from clinical measurements. Pre-pregnancy BMI was calculated as weight in kilograms divided by height in meters squared (kg/m^2^), and categorized as underweight (BMI ≤ 18.5; 109/886, 12.3%), normal weight (18.5 ≤ BMI < 25; 590/886, 66.6%), overweight (25 ≤ BMI < 30; 149/886, 16.8%), and obese (BMI ≥ 30; 38/886, 4.3%) [10]. For subsequent analyses, these categories were grouped into non-overweight (BMI < 25) and overweight (BMI ≥ 25).

Prepartum weight was measured without shoes and in light clothing using a beam balance scale (RGZ-120, Jiangsu Suhong Medical Instruments Co., Changzhou, China). GWG was calculated as the difference between prepartum weight and pre-pregnancy weight. GWG was categorized as inadequate, adequate, or excessive according to the Institute of Medicine (IOM) guidelines [11]. Specifically, adequate GWG was defined as 12.5–18.0 kg for underweight women, 11.5–16.0 kg for normal weight women, 7.0–11.5 kg for overweight women, and 5.0–9.0 kg for women with obesity. GWG values below or above these ranges were classified as inadequate or excessive, respectively.

### 2.3. Child Anthropometric and BP Measurements

Anthropometric and BP measurements were obtained following standardized protocols throughout the follow-up period, with all instruments calibrated prior to use. Children were assessed without shoes and in light clothing. Measurements were conducted from birth to approximately 9 years of age, with data collected four times during infancy (within the first 12 months), twice annually at ages 2 and 3, and once per year thereafter.

Weight was measured to the nearest 0.1 kg using a digital scale (TCS-60, Tianjin Weighing Apparatus Co., Ltd., Tianjin, China). Recumbent length in infants was measured using a length stadiometer (YSC-2, Beijing Guowangxingda, Beijing, China), while standing height in older children was measured using a portable stadiometer; both instruments were accurate to 0.1 cm. During height assessments, children were instructed to stand upright and barefoot with heels together. Each anthropometric measurement was taken twice; if the discrepancy exceeded 0.1 kg for weight or 0.1 cm for height, a third measurement was obtained, and the two closest values were averaged.

BP was measured using an OMRON electronic sphygmomanometer with an appropriately sized cuff. Participants rested in a seated position for at least five minutes before measurement. Systolic BP (SBP) and diastolic BP (DBP) were measured at least twice, with a five-minute interval between readings. If the discrepancy between consecutive readings exceeded 10 mmHg, additional measurements were taken until the discrepancy between the final two readings was within 10 mmHg. The mean of the two closest readings was used in subsequent analyses. As the diagnosis of hypertension typically requires BP measurements on three separate occasions, and measurements in this study were obtained only at the final survey, only screening for HBP was conducted. HBP screening at the final follow-up was performed according to two standards: the Reference of Screening for Elevated Blood Pressure Among Children and Adolescents Aged 7~18 Years (CN standard) and the 2017 Clinical Practice Guideline for Screening and Management of High Blood Pressure in Children and Adolescents (USA standard) [12,13]. Based on both standards, HBP was defined as SBP and/or DBP at or above the 95th percentile for age, sex, and height.

All data collection methods were non-invasive. Written informed consent was obtained from all participants with parental assistance. This study was approved by the Ethics Committee of Peking University (IRB00001052-19099).

### 2.4. Covariates

Maternal covariates were obtained via self-report at the first antenatal care visit, including maternal age, ethnicity, education level, abortion history, last menstrual period, smoking status, and alcohol consumption. Additional information was extracted from medical records, including age at delivery, breastfeeding status (categorized as exclusive breastfeeding, exclusive formula feeding, or mixed feeding), pregnancy outcomes (mode of delivery), and gestational duration (calculated as the interval between the last menstrual period and the delivery date).

Child-related covariates were collected using a structured questionnaire administered at the final follow-up. These included child age, sex, singleton status (singleton or multiple birth), vegetable intake, and frequency of moderate-intensity physical activity. To ensure data accuracy, questionnaires were completed by the children with parental assistance, acknowledging that some items may have been difficult for younger participants to understand independently.

### 2.5. Statistical Analysis

Continuous variables were compared using Student’s *t*-test, as appropriate. Categorical variables were compared using the chi-squared (*χ*^2^) test.

Children’s BMI at each time point was converted into age- and sex- specific BMI-Z scores. A group-based trajectory model (GBTM) was conducted in Stata 17.0 (StataCorp LLC, College Station, TX, USA) to identify BMI trajectory groups from birth to the final follow-up [14]. Successive BMI Z-scores were modeled using a Tobit model, with age as the time metric. Linear, quadratic and cubic polynomial functions were initially fitted, and the optimal polynomial degree was selected based on Bayesian information criteria (BIC) values. The cubic polynomial model demonstrated the best fit. Subsequently, cubic models with 2 to 5 trajectory groups were estimated, and the final five-group model was selected based on (1) the lowest absolute value of BIC, and (2) an average posterior probability ≥ 0.7 (Table A1).

**Figure 1:**
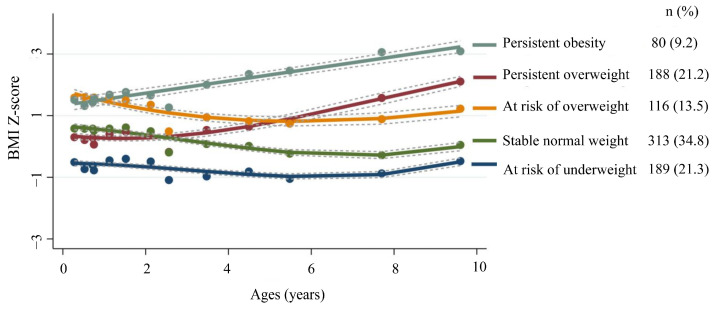
Distinct BMI trajectories in children.

Five distinct BMI trajectories were identified (Figure 1): stable normal weight (*n* = 313, 34.8%), at risk of underweight (*n* = 189, 21.3%), at risk of overweight (*n* = 116, 13.5%), persistent overweight (*n* = 188, 21.2%), and persistent obesity (*n* = 80, 9.2%). The “at risk of underweight” group had BMI-Z scores consistently below −1.28 and was typically observed in premature or low-birth-weight infants. The “stable normal weight” group maintained the BMI-Z scores between −1.28 and +1.04 stably and generally served as the reference group. The “at risk of overweight” group showed BMI-Z scores within the upper normal range (+0.5 to +1.04) with a gradual upward trend. The “persistent overweight” group demonstrated BMI-Z scores exceeding +1.04 with a continued increase (an average annual increase > 0.4). The “persistent obesity” group had BMI-Z scores exceeding +1.88 (P97) at an early age, and continued to rise. This trajectory typically manifests before school age and may contribute to endocrine disorders and cardiovascular complications, imposing a substantial long-term healthcare burden [15,16].

Binomial logistic regression analyses were performed using SPSS version 21.0 (IBM Corp., Armonk, NY, USA) to assess the associations of maternal pre-pregnancy overweight, GWG and BMI trajectories with childhood HBP. The associations were examined in both crude models (Model 1; unadjusted) and adjusted models (Model 2: adjusted for ethnicity, age, children’s diet and exercise habits, maternal breastfeeding, maternal BP during pregnancy, maternal history of hypertension and other diseases, and singleton status). Mediation analysis was conducted using the PROCESS macro (version 4.1) in SPSS. Prior to the mediation analysis, pairwise correlations among the independent variable, mediator, and outcome were examined by binomial logistic regression analyses; mediation analysis was subsequently conducted only if all pairwise correlations were statistically significant. Within this framework, path *a* represents the association between the independent variable and the mediator, path *b* represents the association between the mediator and the outcome, and path *c* (total effect) and path *c*’ (direct effect) represent the total and direct effects of the independent variable on the outcome, respectively. A mediation effect was considered present when both paths a and b were statistically significant (*p* < 0.05). A reduction in the effect size from *c* to *c*’ indicated partial mediation, whereas a non-significant *c*’ indicated complete mediation [17].

Based on different maternal pre-pregnancy overweight status and childhood BMI trajectories, participants were further classified into ten subgroups to estimate the combined effect of maternal pre-pregnancy overweight and childhood BMI trajectories on childhood HBP. The subgroups were: (1) stable normal weight without maternal pre-pregnancy overweight (reference group), (2) stable normal weight with maternal pre-pregnancy overweight, (3) at risk of underweight without maternal pre-pregnancy overweight, (4) at risk of underweight with maternal pre-pregnancy overweight, (5) at risk of overweight without pre-pregnancy overweight, (6) at risk of overweight with pre-pregnancy overweight, (7) persistent overweight without maternal pre-pregnancy overweight, (8) persistent overweight with maternal pre-pregnancy overweight, (9) persistent obesity without maternal pre-pregnancy overweight, and (10) persistent obesity with maternal pre-pregnancy overweight.

All statistical tests were two-sided, and a *p*-value of <0.05 was considered statistically significant.

## 3. Results

A total of 886 children were included in the study, comprising 433 males (mean age 9.12 ± 0.70 years old) and 453 females (mean age 9.08 ± 0.73 years old). At the final follow-up, 14.33% of children were classified as HBP according to the CN standard, and 15% according to the USA standard (Table 1).

### 3.1. Association Between Maternal Pre-Pregnancy Overweight/Obesity and Offspring HBP

**Figure 2:**
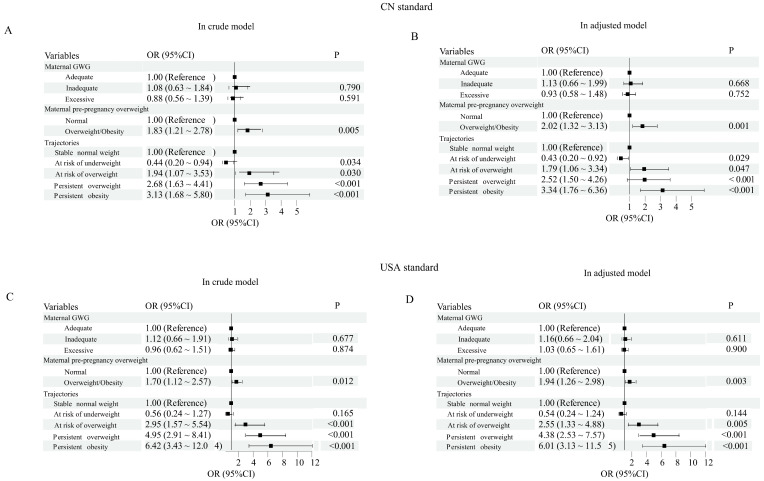
The associations of maternal GWG, maternal pre-pregnancy overweight, and BMI trajectories with childhood HBP. Notes: In (**A**,**B**), the HBP was defined based on the CN standard. In (**A**), the association was assessed by a crude model, and in (**B**), the association was assessed after adjusting for ethnicity, age, children’s diet and exercise habits, maternal breastfeeding, maternal BP during pregnancy, maternal history of hypertension and other diseases, and singleton status. In (**C**,**D**), the HBP was defined based on the USA standard. In (**C**), the association was assessed by a crude model, and in (**D**), the association was assessed after adjusting for ethnicity, age, children’s diet and exercise habits, maternal breastfeeding, maternal BP during pregnancy, maternal history of hypertension and other diseases, and singleton status.

The associations of maternal pre-pregnancy overweight and maternal GWG with childhood HBP were evaluated (Figure 2). Maternal pre-pregnancy overweight was significantly associated with an increased risk of HBP in offspring (RR: 1.83, 95%CI: 1.21–2.78, *p* = 0.001) under the CN standard. However, GWG, whether inadequate or excessive, was not significantly associated with childhood HBP. These findings were consistent in both crude and adjusted models. After adjusting for covariates such as ethnicity, age, children’s diet and exercise habits, and maternal pre-pregnancy overweight, maternal pre-pregnancy overweight remained significantly associated with HBP risk (RR: 2.02, 95%CI: 1.32–3.13, *p* < 0.001), whereas the association between maternal GWG and childhood HBP remained non-significant. Similar associations were observed when applying the USA standard (Figure 2). Sex-stratified analyses showed that maternal pre-pregnancy overweight remained a significant risk factor for HBP in both boys and girls, while GWG continued to show no significant associations (Table A2).

**Table 1:** The characteristics of participants.

	Mean ± SE/*N* (%)			*t*/*χ*^2^	*p*
Variables	Total (*N* = 886)	Male (*N* = 433)	Female (*N* = 453)		
Child factors at last follow-up					
Age (years)	9.11 ± 0.71	9.12 ± 0.73	9.08 ± 0.70	0.80	0.422
Height (cm)	141.07 ± 7.57	141.44 ± 7.46	140.71 ± 7.67	1.43	0.153
Weight (kg) *	38.31 ± 11.03	40.50 ± 12.06	36.21 ± 9.49	5.90	<0.001
SBP (mmHg) *	108.52 ± 10.86	110.07 ± 11.05	107.05 ± 10.47	4.17	<0.001
DBP (mmHg)	63.98 ± 6.68	63.92 ± 6.80	64.04 ± 6.56	−0.22	0.796
HBP by CN standard					
Normal BP (%)	759 (85.7)	366 (84.5)	393 (86.7)	0.90	0.341
HBP (%)	127 (14.3)	67 (15.5)	60 (13.3)		
HBP by USA standard *					
Normal BP (%)	753 (85.0)	350 (80.8)	403 (88.9)	13.34	<0.001
HBP (%)	133 (15.0)	83 (19.2)	50 (10.1)		
Vegetable intake					
7 days per week (%)	244 (27.9)	110 (25.8)	134 (29.8)	1.70	0.190
<7 days per week (%)	632 (72.1)	316 (74.2)	316 (70.2)		
Fried food intake					
0 times per week (%)	269 (30.4)	132 (30.5)	137 (30.2)	0.01	0.941
>0 times per week (%)	617 (69.6)	301 (69.5)	316 (69.8)		
Sweet sugary beverages					
Yes (%)	501 (57.2)	252 (59.2)	249 (55.3)	1.31	0.252
No (%)	375 (42.8)	174 (40.8)	201 (44.7)		
moderate-intensity physical activity, per week					
>3 days (%)	574 (64.8)	279 (64.4)	295 (65.1)	0.05	0.831
≤3 days (%)	312 (35.2)	154 (35.6)	158 (34.9)		
Maternal factors					
Breastfeeding					
Exclusively (%)	504 (58.3)	243 (57.3)	261 (59.3)	1.90	0.391
Exclusively formula (%)	128 (14.8)	70 (16.5)	58 (13.2)		
Mixed	232 (26.9)	111 (26.2)	121 (27.5)		
Pre-pregnancy BMI					
Normal weight (%)	699 (78.9)	344 (79.4)	355 (78.4)	0.04	0.852
Overweight (%)	187 (21.1)	89 (20.6)	98 (21.6)		
GWG					
Inadequate (%)	109 (12.3)	51 (11.8)	58 (12.8)	2.86	0.244
Adequate (%)	253 (28.6)	135 (31.2)	118 (26.1)		
Excessive (%)	524 (59.1)	247 (57.0)	277 (61.1)		
Pre-pregnancy BP					
Normal BP (%)	681 (76.9)	332 (76.7)	349 (77.0)	0.02	0.909
HBP (%)	205 (23.1)	101 (23.3)	104 (23.0)		
History of hypertension					
Yes (%)	25 (2.9)	9 (2.1)	16 (3.6)	1.76	0.198
No (%)	839 (97.1)	415 (97.9)	424 (96.4)		
History of other diseases					
Yes (%)	6 (0.7)	5 (1.2)	1 (0.2)	2.87	0.091
No (%)	880 (99.3)	428 (98.8)	452 (99.8)		
Singleton status					
Singleton (%)	867 (97.9)	427 (98.6)	440 (97.1)	2.32	0.131
Multiple birth (%)	19 (2.1)	6 (1.4)	13 (2.9)		

Note: The variables were presented in all participants (the first column), males (the second column), and females (the third column), respectively. HBP were screened based on the CN and USA standards. The comparison of variables was conducted in males and females. The * meant the differences in the variable between different sexes were significant.

### 3.2. Impact of Offspring BMI Trajectories on HBP

The associations of BMI trajectories with childhood HBP were also investigated (Figure 2). In the crude model, ‘at risk of overweight’ (RR: 1.94, 95%CI: 1.07–3.53, *p* = 0.034), “persistent overweight” (RR: 2.68, 95%CI: 1.63–4.41, *p* < 0.001) and “persistent obesity” (RR: 3.13, 95%CI: 1.69–5.80, *p* < 0.001) trajectories were associated with the elevated risk of HBP. These associations remained significant after adjusting for covariates such as ethnicity, age, and children’s diet and exercise habits. Results based on USA standards were consistent with those based on the CN standard. In sex-stratified analyses, both persistent overweight and persistent obesity trajectories were significantly associated with elevated risk of HBP in both boys and girls (Table A2).

### 3.3. Role of Offspring BMI Trajectories in the Association Between Maternal Pre-Pregnancy Overweight/Obesity and Childhood HBP

**Figure 3:**
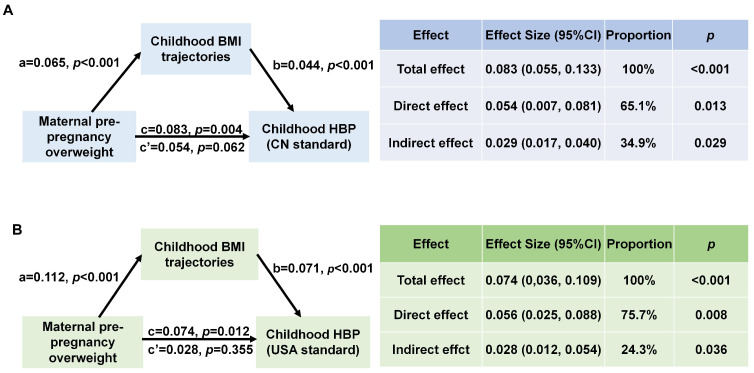
The probable modulating effect size and the proportion of childhood BMI trajectories on the association between maternal pre-pregnancy overweight and childhood HBP. Notes: In the (**A**), HBP was screened based on the CN standard. In the (**B**), HBP was screened based on the USA standard.

The effect of maternal pre-pregnancy overweight on offspring’s BMI trajectories was further investigated (Table 2). Maternal pre-pregnancy overweight status was treated as the independent variable, and “without maternal pre-pregnancy overweight” served as the reference group. The different BMI trajectories were treated as distinct outcomes, with the stable normal weight serving as the reference group. Maternal pre-pregnancy overweight was associated with an increased likelihood of children following a persistent overweight trajectory (RR: 1.84, 95%CI: 1.26–2.69, *p* < 0.001) or a persistent obesity trajectory (RR: 3.88, 95%CI: 2.16–6.97, *p* < 0.001). These associations were consistent across boys and girls (Table A3). Given that BMI trajectories were associated with both the maternal pre-pregnancy overweight and childhood HBP, the mediation effect model was conducted. Results indicated that BMI trajectories may be associated with the relationship between maternal pre-pregnancy overweight and offspring HBP (Figure 3) (*p* < 0.05). The direct association between maternal pre-pregnancy overweight and childhood HBP was estimated at 0.054 (95%CI: 0.007–0.081, *p* < 0.05, the proportion: 65.1%), and the indirect association was estimated at 0.029 (95%CI: 0.017–0.040, *p* < 0.05, the proportion: 34.9%). Similar results were observed when applying the USA HBP standard.

### 3.4. Joint Effects of Maternal Pre-Pregnancy Overweight and Offspring BMI Trajectories on Offspring’s HBP

To explore the joint effects, participants were regrouped by maternal pre-pregnancy weight status and offspring BMI trajectories (Table 3). Maternal pre-pregnancy overweight was significantly associated with elevated HBP risk across all BMI trajectories (all *p* < 0.05), with particularly high relative risks observed in children within the “at risk of overweight” (RR: 3.67, 95%CI: 1.78–7.59, *p* < 0.001), “persistent overweight” (RR: 3.35, 95%CI: 1.57–7.12, *p* = 0.002), and “persistent obesity” (RR: 4.02, 95%CI: 1.60–10.08, *p* = 0.003) trajectories. Comparable results were observed when applying the USA standard (Table A4). These associations remained significant after adjusting for covariates. We further examined the associations between maternal pre-pregnancy overweight and offspring HBP within each BMI trajectory group (Table 4), using children without maternal pre-pregnancy overweight as the reference group. In the “at risk of overweight” (RR: 2.58, 95%CI: 1.66–3.70, *p* = 0.007) and “persistent overweight” (RR: 2.03, 95%CI: 1.57–2.46, *p* = 0.043) trajectory groups, maternal pre-pregnancy overweight was associated with an elevated risk of childhood HBP. These associations remained stable and significant after adjusting for covariates. Similar results were obtained based on the USA standard (Table A5).

## 4. Discussion

This exploratory study found that maternal pre-pregnancy overweight was linked to an elevated risk of childhood HBP in offspring, and that offspring BMI trajectories may be associated with the linkage. Children in the persistent overweight and persistent obesity trajectory groups with maternal pre-pregnancy overweight showed a higher risk of HBP. These findings suggest that rapid BMI increase and maternal pre-pregnancy overweight may have a combined effect on childhood HBP.

Maternal pre-pregnancy overweight is related to the occurrence of various adverse health outcomes in offspring. It has been shown to increase the risk of overweight and obesity in both childhood and adulthood, as well as the risk of type 1 diabetes [18,19]. In addition, maternal pre-pregnancy overweight has been linked to an elevated risk of cardiovascular disease in adulthood, including hypertension and dyslipidemia [20,21]. However, existing research has primarily focused on the impact of maternal pre-pregnancy overweight/obesity on cardiovascular health in adulthood, while its impact on cardiovascular health in childhood has received less attention. The present findings suggest that maternal pre-pregnancy overweight may not only increase the risk of HBP in adulthood but also be associated with an elevated risk in childhood.

Maternal pre-pregnancy overweight may contribute to increasing BMI, persistently high BMI, and overweight/obesity in offspring [22,23,24]. In this study, it was also associated with BMI trajectories characterized by persistent overweight and persistent obesity. Long-term rapid weight gain during childhood is an important risk factor for HBP in children and adolescents [25], potentially exerting cumulative effects through mechanisms such as inflammation or oxidative stress [26,27,28]. One longitudinal cohort study tracking BMI from ages 6 to 17 years found that adolescents in the “high BMI increasing” and “highest BMI increasing” trajectories had a significantly elevated risk of HBP, in both males and females [29]. Another study also reported that rapidly increasing BMI trajectories during childhood were associated with a higher risk of HBP in adolescence [30]. In our study, rapidly increasing BMI was similarly associated with an elevated risk of HBP in children, which aligns with previous reports. Notably, most previous studies began tracking BMI around 5–6 years of age, whereas the present study included measurements from birth, allowing for a more comprehensive assessment of the cumulative impact of early-life BMI trajectories on childhood HBP.

In this study, BMI trajectories may be associated with the relationship between maternal pre-pregnancy overweight and childhood HBP. Maternal pre-pregnancy overweight may be linked to childhood HBP both directly and indirectly by early-life BMI growth patterns. This highlights the important role of BMI increase in early life on children’s BP [31]. Collectively, the effects of BMI on childhood HBP may partly originate from maternal pre-pregnancy weight status. In addition to the direct effect, maternal pre-pregnancy overweight may also contribute to rapid BMI increases in offspring, which may subsequently elevate HBP risk.

Subgroup analyses found that children in increasing BMI trajectories (i.e., persistent overweight and persistent obesity) with maternal pre-pregnancy overweight had higher relative risks, suggesting a combined effect of rapid BMI increase and maternal pre-pregnancy overweight on childhood HBP. Given that childhood HBP may lead to adult hypertension and contribute to early target organ damage, including the heart, brain, and kidneys, and that early-stage HBP is often reversible, early identification of children at risk is crucial [32]. These findings may help identify priority target populations for prevention and intervention.

This study has several limitations. First, no association was observed between maternal GWG and offspring HBP, which may be due to the limited sample size. Future studies with larger cohorts are needed to enable more detailed subgroup analyses. In addition, samples from the same city may also affect the accuracy and generalizability. Second, body composition (e.g., fat and muscle mass distribution), which is known to be associated with cardiovascular health, was not assessed [33]. Future research should incorporate direct measures of body composition to provide a more comprehensive understanding of early growth patterns and cardiovascular outcomes. Third, HBP in this study was defined based on a single measurement, which may reduce accuracy. Hypertension diagnosis typically requires measurements on three separate occasions [34]. Therefore, the study was conducted based on screening for HBP rather than diagnosis, which may reduce the accuracy. In the future, the frequency of BP measurements in the follow-up visit should be increased. Finally, the selection bias resulting from sample selection and exclusion criteria, along with the reliance on self-reported covariates, may influence the study results and reduce the external validity of the study.

## 5. Conclusions

Increasing BMI trajectories in early life and maternal pre-pregnancy overweight are associated with an elevated risk of childhood HBP. Therefore, maternal pre-pregnancy overweight is also related to offspring BMI trajectories. BMI trajectories seemed to exert a probable modulating effect on maternal pre-pregnancy overweight and offspring HBP. Maternal pre-pregnancy BMI may influence childhood HBP both directly and indirectly through its effect on offspring BMI trajectories. Furthermore, maternal pre-pregnancy overweight and increasing BMI in offspring may exert a combined effect on the offspring’s HBP. These findings suggest that children with increasing BMI trajectories and maternal pre-pregnancy overweight may be considered the key target population for childhood HBP prevention and intervention.

## Figures and Tables

**Table 2:** The association between maternal pre-pregnancy and offspring’s BMI trajectories.

Variables	Model 1			Model 2		
	Normal (Ref)	Maternal Pre-Pregnancy Overweight	*p*	Normal (Ref)	Maternal Pre-Pregnancy Overweight	*p*
	RR	RR (95%CI)		RR	RR (95%CI)	
Model 1						
Stable normal weight (ref)	1			1		
At risk of underweight		0.63 (0.35, 1.30)	0.594		0.79 (0.32, 1.96)	0.609
At risk of overweight		2.77 (1.66, 4.62)	<0.001		1.15 (0.69, 1.92)	0.601
Persistent overweight		1.87 (1.29, 2.73)	<0.001		4.22 (2.01, 8.82)	<0.001
Persistent obesity		3.43 (1.93, 6.09)	<0.001		3.72 (1.65, 8.35)	0.001

Note: Maternal pre-pregnancy overweight status was treated as the independent variable, and “without maternal pre-pregnancy overweight” served as the reference group. The different BMI trajectories were distinct outcomes, and the stable normal weight served as the reference group. Model 1 was a crude model. Model 2 was adjusted for ethnicity, age, children’s diet and exercise habits, maternal breastfeeding, maternal BP during pregnancy, maternal history of hypertension and other diseases, and singleton status.

**Table 3:** The subgroup analysis based on maternal pre-pregnancy overweight and BMI trajectories was conducted to examine the joint effect (CN standard).

The Subgroups	Model 1		Model 2	
	RR (95%CI)	*p*	RR (95%CI)	*p*
Without Maternal Pre-pregnancy Overweight				
Stable normal weight	1		1	
At risk of underweight	2.01 (0.84, 4.78)	0.115	2.08 (0.86, 5.04)	0.106
At risk of overweight	0.56 (0.25, 1.23)	0.148	0.54 (0.24, 1.19)	0.127
Persistent overweight	—	—	—	—
Persistent obesity	1.98 (1.05, 4.09)	0.066	1.71 (0.79, 3.70)	0.176
Maternal Pre-pregnancy Overweight				
Stable normal weight	2.77 (1.14, 6.73)	0.025	2.81 (1.14, 6.97)	0.025
At risk of underweight	2.83 (1.57, 5.09)	0.001	2.54 (1.36, 4.74)	0.003
At risk of overweight	3.67 (1.78, 7.59)	<0.001	3.79 (1.78, 8.04)	0.001
Persistent overweight	3.35 (1.57, 7.12)	0.002	3.56 (1.61, 7.86)	0.002
Persistent obesity	4.02 (1.60, 10.08)	0.003	4.27 (1.67, 10.93)	0.002

Note: Model 1 was a crude model. Model 2 was adjusted for ethnicity, age, children’s diet and exercise habits, maternal breastfeeding, maternal BP during pregnancy, maternal history of hypertension and other diseases, and singleton status. Stable normal weight without maternal pre-pregnancy overweight served as the reference group. The “—“ means that the sample size of this group is insufficient, and statistical analysis cannot be conducted. HBP was defined based on the CN standard.

**Table 4:** The association between maternal pre-pregnancy with HBP in each BMI trajectory (CN standard).

Subgroups	Model 1		Model 2	
	RR (95%CI)	*p*	RR (95%CI)	*p*
Stable normal weight				
Without maternal Pre-pregnancy Overweight	1		1	
Maternal Pre-pregnancy Overweight	1.79 (1.16, 3.73)	0.026	1.66 (0.96, 2.36)	0.064
At risk of underweight				
Without maternal Pre-pregnancy Overweight	1		1	
Maternal Pre-pregnancy Overweight	1.06 (0.86, 1.40)	0.169	1.14 (0.91, 1.53)	0.138
At risk of overweight				
Without maternal Pre-pregnancy Overweight	1		1	
Maternal Pre-pregnancy Overweight	2.58 (1.66, 3.70)	0.007	2.24 (1.45, 2.98)	0.011
Persistent overweight				
Without maternal Pre-pregnancy Overweight	1		1	
Maternal Pre-pregnancy Overweight	—	—	—	—
Persistent obesity				
Without maternal Pre-pregnancy Overweight	1		1	
Maternal Pre-pregnancy Overweight	2.03 (1.57, 2.46)	0.004	2.16 (1.55, 2.79)	0.016

Note: Model 1 was a crude model. Model 2 was adjusted for ethnicity, age, children’s diet and exercise habits, maternal breastfeeding, maternal BP during pregnancy, maternal history of hypertension and other diseases, and singleton status. In each trajectory, the group without maternal pre-pregnancy overweight served as the reference group. The “—“ means that the sample size of this group is insufficient, and statistical analysis cannot be conducted. HBP was defined based on the CN standard.

## Data Availability

The data in this study were collected through on-site investigations and questionnaire surveys.

## Abbreviations

BP	Blood Pressure
HBP	High Blood Pressure
BMI	Body Mass Index
GWG	gestational weight gain
GBTM	Group-Based Trajectory Model

## Appendix A

**Table A1:** Group-based trajectory model (GBTM) results of the model-fitting process.

No. of Class	Polynomial Degree	Log-Likelihood	BIC (*N* = 1056)	BIC (*N* = 6592)	Participants per Class (%)	Mean Posterior Probabilities
1	L	−9994.15	−10,001.59	−10,004.34	100	
1	Q	−9951.07	−9961	−9964.66	100	
1	C	−9950.31	−9962.71	−9967.29	100	
2	L/L	−9155.33	−9170.22	−9175.71	56.07/43.93	0.95/0.94
2	Q/Q	−9105.91	−9098.59	−9078.74	55.15/44.85	0.95/0.95
2	C/C	−9072.36	−9097.17	−9106.32	53.16/46.84	0.95/0.95
3	L/L/L	−8976.98	−8999.31	−9007.54	33.59/46.08/20.33	0.93/0.92/0.92
3	Q/Q/Q	−8888.71	−8918.48	−8929.46	34.32/45.64/20.04	0.93/0.93/0.92
3	C/C/C	−8871.91	−8909.13	−8922.85	32.42/41.29/21.29	0.94/0.93/0.93
4	L/L/L/L	−8902.24	−8932.02	−8943.00	20.95/39.86/31.78/7.41	0.92/0.89/0.88/0.89
4	Q/Q/Q/Q	−8789.07	−8828.76	−8843.4	23.17/38.58/30.05/8.17	0.92/0.89/0.88/0.90
4	C/C/C/Q	−8759.2	−8928.2	−8827.13	28.94/33.46/28.07/9.52	0.92/0.91/0.89/0.90
5	L/L/L/L/L	−8890.98	−12,315.85	−8941.92	26.38/19.31/38.52/8.30/7.50	0.91/0.87/0.81/0.86/0.93
5	Q/Q/Q/Q/Q	−8741.57	−8791.19	−8809.49	19.76/36.12/27.96/8.29/7.88	0.91/0.87/0.81/0.86/0.93
5	C/C/C/C/L	−8671.49	−8733.52	−8756.39	19.35/36.69/9.46/26.31/8.19	0.91/0.87/0.82/0.87/0.93

**Table A2:** The associations between maternal pre-pregnancy overweight, maternal GWG, offspring’s BMI trajectories, and offspring’s HBP.

Variables	In Boys				In Girls			
	HBP (CN)		HBP (USA)		HBP (CN)		HBP (USA)	
	RR (95%CI)	*p*	RR (95%CI)	*p*	RR (95%CI)	*p*	RR (95%CI)	*p*
Model 1								
Maternal pre-pregnancy BMI								
Non-overweight (Ref)	1		1		1		1	
overweight/obesity	1.53 (1.24, 2.78)	0.047	1.29 (1.33, 2.28)	0.038	2.21 (1.23, 3.97)	0.008	2.51 (1.35, 4.68)	0.004
Maternal GWG								
Inadequate	1.38 (0.64, 2.97)	0.409	1.17 (0.58, 2.37)	0.667	0.84 (0.39, 1.79)	0.643	1.12 (0.49, 2.56)	0.784
Adequate (Ref)	1		1		1		1	
Excessive	1.28 (0.68, 2.41)	0.453	1.22 (0.69, 2.16)	0.503	0.6 (0.32, 1.15)	0.124	0.74 (0.36, 1.53)	0.421
Offspring’s BMI trajectories								
Stable normal weight (Ref)	1		1		1		1	
At risk of underweight	0.33 (0.09, 1.18)	0.088	0.36 (0.10, 1.30)	0.119	0.53 (0.20, 1.37)	0.189	0.79 (0.27, 2.34)	0.67
At risk of overweight	1.92 (0.82, 4.48)	0.134	2.31 (0.99, 5.42)	0.053	1.93 (0.81, 4.57)	0.135	3.49 (1.36, 8.95)	0.009
Persistent overweight	2.3 (1.11, 4.75)	0.024	3.95 (1.93, 8.07)	<0.001	3.29 (1.62, 6.67)	0.001	5.53 (2.46, 12.43)	<0.001
Persistent obesity	3.59 (1.54, 8.39)	0.003	6.85 (2.99, 15.72)	<0.001	2.42 (1.13, 6.33)	0.041	4.38 (1.56, 12.31)	0.005
Model 2								
Maternal pre-pregnancy BMI								
Non-overweight (Ref)	1		1		1		1	
overweight/obesity	1.75 (1.12, 3.32)	0.046	1.55 (1.08, 2.83)	0.033	2.14 (1.17, 3.92)	0.013	2.42 (1.28, 4.59)	0.007
Maternal GWG								
Inadequate	1.44 (0.63, 3.31)	0.384	1.01 (0.47, 2.18)	0.979	0.91 (0.41, 2.00)	0.806	1.25 (0.53, 2.95)	0.607
Adequate (Ref)	1		1		1		1	
Excessive	1.23 (0.63, 2.41)	0.547	1.11 (0.61, 2.02)	0.732	0.63 (0.32, 1.23)	0.178	0.78 (0.37, 1.66)	0.526
Offspring’s BMI trajectories								
Stable normal weight (Ref)	1		1		1		1	
At risk of underweight	0.31 (0.08, 1.15)	0.08	0.35 (0.09, 1.30)	0.117	0.51 (0.20, 1.34)	0.174	0.78 (0.26, 2.35)	0.665
At risk of overweight	1.55 (0.62, 3.84)	0.346	1.97 (0.80, 4.82)	0.138	1.78 (0.73, 4.31)	0.204	3.09 (1.18, 8.11)	0.022
Persistent overweight	1.89 (1.28, 4.04)	0.013	3.53 (1.69, 7.37)	0.001	3.14 (1.51, 6.54)	0.002	5.39 (2.34, 12.40)	<0.001
Persistent obesity	3.24 (1.32, 7.93)	0.010	5.77 (2.42, 13.76)	<0.001	2.62 (1.26, 7.11)	0.039	4.84 (1.66, 14.13)	0.004

Note: Model 1 was a crude model. Model 2 was adjusted for ethnicity, age, children’s diet and exercise habits, maternal breastfeeding, maternal BP during pregnancy, maternal history of hypertension and other diseases, and singleton status. Stable normal weight, non-maternal pre-pregnancy overweight, and maternal GWG adequate served as the reference groups, respectively.

**Table A3:** The association between maternal pre-pregnancy overweight and offspring’s BMI trajectories in boys and girls.

Variables	Model 1			Model 2		
	Without Maternal Pre-Pregnancy Overweight (Ref)	Maternal Pre-Pregnancy Overweight	*p*	Without Maternal Pre-Pregnancy Overweight (Ref)	Maternal Pre-Pregnancy Overweight	*p*
In boys						
Stable normal weight (ref)	1			1		
At risk of underweight		0.79 (0.32, 1.96)	0.609		0.77 (0.31, 1.94)	0.579
At risk of overweight		1.15 (0.69, 1.92)	0.601		1.05 (0.62, 1.80)	0.847
Persistent overweight		4.22 (2.01, 8.82)	<0.001		4.81 (2.25, 10.31)	<0.001
Persistent obesity		3.72 (1.65, 8.35)	0.001		4.19 (1.83, 9.61)	0.001
In girls						
Stable normal weight (ref)	1			1		
At risk of underweight		0.49 (0.23, 1.03)	0.059		0.58 (0.27, 1.25)	0.162
At risk of overweight		1.63 (0.78, 3.39)	0.193		1.77 (0.84, 3.74)	0.136
Persistent overweight		3.38 (1.97, 5.79)	<0.001		3.48 (2.01, 6.01)	<0.001
Persistent obesity		2.72 (1.22, 6.07)	0.015		3.45 (1.48, 8.04)	0.004

Note: Maternal pre-pregnancy overweight status was treated as the independent variable, and “without maternal pre-pregnancy overweight” served as the reference group. The different BMI trajectories were distinct outcomes, and the stable normal weight served as the reference group. Model 1 was a crude model. The model 2 was adjusted for ethnicity, age, children’s diet and exercise habits, maternal breastfeeding, maternal BP during pregnancy, maternal history of hypertension and other diseases, and singleton status.

**Table A4:** The subgroup analysis based on maternal pre-pregnancy overweight and BMI trajectories was conducted to examine the joint effect (USA standard).

**The Subgroups**	**Model 1**			**Model 2**		
	**RR**	**95%CI**	***p***	**RR**	**95%CI**	***p***
Without Maternal Pre-pregnancy Overweight						
Stable normal weight	1			1		
At risk of underweight	2.29	0.84, 6.27	0.106	2.29	0.84, 6.27	0.106
At risk of overweight	0.71	0.29, 1.67	0.42	0.71	0.29, 1.67	0.42
Persistent overweight	—	—	—	—	—	—
Persistent obesity	3.12	1.45, 6.72	0.004	3.12	1.45, 6.72	0.004
Maternal Pre-pregnancy Overweight						
Stable normal weight	2.67	0.97, 7.36	0.058	2.67	0.97, 7.36	0.058
At risk of underweight	4.77	2.50, 9.11	<0.001	4.77	2.50, 9.11	<0.001
At risk of overweight	5.93	2.70, 13.00	<0.001	5.93	2.70, 13.00	<0.001
Persistent overweight	5.78	2.57, 13.01	<0.001	5.78	2.57, 13.01	<0.001
Persistent obesity	9.38	3.74, 20.55	<0.001	9.38	3.74, 20.55	<0.001

Note: Model 1 was a crude model. Model 2 was adjusted for ethnicity, age, children’s diet and exercise habits, maternal breastfeeding, maternal BP during pregnancy, maternal history of hypertension and other diseases, and singleton status. Stable normal weight without maternal pre-pregnancy served as the reference group. The “—“ means that the sample size of this group is insufficient, and statistical analysis cannot be conducted. HBP was defined based on the USA standard.

**Table A5:** The association between maternal pre-pregnancy with HBP in each BMI trajectory (USA standard).

**Subgroups**	**Model 1**			**Model 2**		
	**RR**	**95%CI**	***p***	**RR**	**95%CI**	***p***
Stable normal weight						
Without maternal Pre-pregnancy Overweight	1			1		
Maternal Pre-pregnancy Overweight	1.53	0.79, 2.45	0.241	1.46	0.85, 1.93	0.186
At risk of underweight						
Without maternal Pre-pregnancy Overweight	1			1		
Maternal Pre-pregnancy Overweight	1.21	0.90, 1.69	0.098	1.26	0.94, 1.73	0.065
At risk of overweight						
Without maternal Pre-pregnancy Overweight	1			1		
Maternal Pre-pregnancy Overweight	2.32	1.45, 3.47	0.003	2.56	1.73, 3.62	0.008
Persistent overweight						
Without maternal Pre-pregnancy Overweight	1			1		
Maternal Pre-pregnancy Overweight	—	—	—	—	—	—
Persistent obesity						
Without maternal Pre-pregnancy Overweight	1			1		
Maternal Pre-pregnancy Overweight	2.14	1.49, 2.70	0.023	2.27	1.55, 2.79	0.014

Note: Model 1 was a crude model. Model 2 was adjusted for ethnicity, age, children’s diet and exercise habits, maternal breastfeeding, maternal BP during pregnancy, maternal history of hypertension and other diseases, and singleton status. Stable normal weight without maternal pre-pregnancy served as the reference group. The “—“ means that the sample size of this group is insufficient, and statistical analysis cannot be conducted. HBP was defined based on the USA standard.
